# Access to insulin: applying the concept of security of supply to medicines

**DOI:** 10.2471/BLT.18.217612

**Published:** 2019-03-26

**Authors:** David Beran, Zafar Mirza, Jicui Dong

**Affiliations:** a Division of Tropical and Humanitarian Medicine, University of Geneva, Rue Gabrielle-Perret-Gentil 6, CH-1211 Geneva 14, Switzerland.; b Department of Health System Development, World Health Organization Regional Office for the Eastern Mediterranean, Cairo, Egypt.; c Department of Essential Medicines and Health Products, World Health Organization, Geneva, Switzerland.

## Abstract

Security of supply of medicines is fundamental to ensure health for all. Furthermore, improving access to medicines is included in sustainable development goal 3. However, the concept of security of supply has mostly been applied to food, water and energy. Diversity of supply, vulnerability to disruption, expenditure, infrastructure, stability of exporting countries, ownership of production, price stability, access and equity, affordability, intellectual property, safety and reliability of supply, and countries’ capacity to adapt to market changes are all elements of security of supply. Based on these elements, we assessed security of supply for insulin, since access to insulin is a global problem. We found that three multinational companies, in Denmark, France and Germany, control 99% of the value of the global insulin market. Prices and affordability of insulin and access to it vary considerably between countries. Some countries are vulnerable to insulin shortage because they import insulin from only one source. Many countries spend large amounts of money on insulin and costs are increasing. Some countries lack an adequate infrastructure for procurement, supply chain management and distribution of insulin. Applying the security of supply concept to insulin showed that diversification of suppliers needs to be fostered. Global health actors should adopt a security of supply approach to identify medicines that are susceptible to supply issues and address this concern by strategic promotion of local production, strengthening regulatory harmonization, and adding local products to the World Health Organization’s programme on prequalification of medicines.

## Résumé

La sécurité de l'approvisionnement en médicaments est fondamentale pour garantir une bonne santé pour tous. De plus, l'amélioration de l'accès aux médicaments figure dans l'Objectif de développement durable n°3. Or, le concept de sécurité d'approvisionnement a jusqu'à présent été principalement appliqué aux aliments, aux boissons et à l'énergie. La diversité d'approvisionnement, la vulnérabilité face aux perturbations de la chaîne d'approvisionnement, les infrastructures, la stabilité des pays exportateurs, la propriété des moyens de production, la stabilité des prix, l'accès et l'équité, l'accessibilité des prix, la propriété intellectuelle, la sûreté et la fiabilité de l'approvisionnement ainsi que la capacité des pays à s'adapter aux évolutions du marché sont autant de notions inhérentes à la sécurité d'approvisionnement. À partir de ces éléments, nous avons évalué la sécurité d'approvisionnement pour l'insuline, étant donné que l'accès à l'insuline constitue un enjeu mondial. Nous avons constaté que trois multinationales (au Danemark, en France et en Allemagne) contrôlent 99% de la valeur du marché mondial de l'insuline. Les prix, leur accessibilité ainsi que l'accès à l'insuline varient considérablement d'un pays à un autre. Certains pays sont vulnérables face aux pénuries d'insuline, car ils n'importent l'insuline qu'auprès d'une seule source. De nombreux pays dépensent énormément d'argent pour l'insuline, et les coûts sont en augmentation. Certains pays n'ont pas d'infrastructures appropriées pour les achats, la gestion de la chaîne d'approvisionnement et la distribution de l'insuline. L'application du concept de sécurité d'approvisionnement à l'insuline nous a montré que la diversification des fournisseurs doit être encouragée. Les acteurs mondiaux de la santé devraient adopter une approche de sécurité d'approvisionnement pour identifier les médicaments susceptibles de connaître des problèmes d'approvisionnement et répondre à cet enjeu par la promotion stratégique d'une production locale, le renforcement de l'harmonisation réglementaire et l'ajout de produits locaux dans le programme de l'Organisation mondiale de la Santé sur la préqualification des médicaments.

## Resumen

La seguridad en el suministro de medicamentos es fundamental para garantizar la salud de todos. Además, la mejora del acceso a los medicamentos está incluida en el objetivo de desarrollo sostenible 3. Sin embargo, el concepto de seguridad en el suministro se ha aplicado principalmente a los alimentos, el agua y la energía. La diversidad del suministro, la vulnerabilidad a las perturbaciones, el gasto, la infraestructura, la estabilidad de los países exportadores, la propiedad de la producción, la estabilidad de los precios, el acceso y la equidad, la asequibilidad, la propiedad intelectual, la seguridad y la fiabilidad del suministro y la capacidad de los países para adaptarse a los cambios del mercado son todos elementos que se incluyen en la seguridad en el suministro. Con base en estos elementos, se ha evaluado la seguridad del suministro de insulina, ya que el acceso a esta es un problema mundial. Se ha descubierto que tres empresas multinacionales, en Dinamarca, Francia y Alemania, controlan el 99 % del valor de mercado mundial de insulina. Los precios y la asequibilidad de la insulina y el acceso a ella varían considerablemente de un país a otro. Algunos países son vulnerables a la escasez de insulina porque la importan de una sola fuente. Muchos países gastan grandes cantidades de dinero en insulina y los costes aumentan. Algunos países carecen de una infraestructura adecuada para la adquisición, la gestión de la cadena de suministro y la distribución de insulina. La aplicación del concepto de seguridad en el suministro a la insulina demostró que es necesario fomentar la diversificación de los proveedores. Los agentes de la salud mundial deberían adoptar un enfoque de seguridad en el suministro para identificar los medicamentos que son susceptibles de problemas de suministro y abordar esta preocupación mediante la promoción estratégica de la producción local, el fortalecimiento de la armonización de los reglamentos y la incorporación de los productos locales al programa de la Organización Mundial de la Salud sobre la precalificación de los medicamentos.

## ملخص

يعد تأمين الإمداد بالأدوية أمرًا أساسيًا لضمان الصحة للجميع. علاوة على ذلك، فإن تحسين الحصول على الأدوية يتضمن الهدف 3 من أهداف التنمية المستدامة. ومع ذلك، فقد تم تطبيق مفهوم تأمين الإمداد في الغالب على الغذاء والماء والطاقة. تنوع الإمداد، وإمكانية التعرض للانقطاع، والإنفاق، والبنية التحتية، واستقرار البلدان المصدرة، وملكية الإنتاج، واستقرار الأسعار، وإمكانية الحصول عليه بإنصاف، والقدرة على تحمل التكاليف، والملكية الفكرية، وسلامة وموثوقية الإمداد، وقدرة البلدان على التكيف مع تغيرات السوق، تمثل جميعها عناصر لأمن الإمداد. واستنادًا إلى هذه العناصر، قمنا بتقييم أمان الإمداد بالأنسولين، نظرًا لأن الحصول عليه يمثل مشكلة عالمية. وجدنا أن ثلاث شركات متعددة الجنسيات في الدنمرك وفرنسا وألمانيا، تسيطر على 99٪ من قيمة سوق الأنسولين العالمي. تختلف أسعار الأنسولين، والقدرة على تحملها، وإمكانية الوصول إليها، اختلافًا كبيرًا بين البلدان. بعض البلدان معرضة لنقص الأنسولين نظراً لأنها تستورد الأنسولين من مصدر واحد فقط. تنفق العديد من البلدان مبالغ طائلة على الأنسولين، ولا زالت التكاليف في صعود. تفتقر بعض البلدان إلى بنية تحتية مناسبة للمشتريات ولإدارة سلسلة التوريد وتوزيع الأنسولين. إن تطبيق مفهوم أمان الإمداد على الأنسولين أظهر أن تنويع الموردين يحتاج إلى رعاية. ينبغي أن تعتمد الجهات الصحية العالمية الفاعلة نهجاً تأمين الإمداد، لتحديد الأدوية المعرضة لقضايا الإمداد، ومعالجة هذا الشأن من خلال الترويج الاستراتيجي للإنتاج المحلي، وتعزيز التنسيق التنظيمي، وإضافة منتجات محلية إلى برنامج منظمة الصحة العالمية الخاص بالتأهيل المسبق للأدوية.

## 摘要

药品供应安全是确保人人享有健康的基础。此外，增加获取药物的机会已纳入可持续发展目标 3。然而，供应安全概念主要应用于食品、水和能源。供应的多样性、脆弱性、支出、基础设施、出口国稳定性、产品所有权、价格稳定性、获取与公平、可负担性、知识产权、供应安全性和可靠性以及各国适应市场变化的能力，都是供应安全的要素。基于这些要素，我们对胰岛素供应的安全性进行了评估，因为对胰岛素的获取是一个全球问题。结果表明，丹麦、法国和德国的三家跨国公司控制着全球 99% 的胰岛素市场份额。胰岛素的价格、可负担性和可得性在各国之间有显著差异。某些国家胰岛素进口渠道只有一个，因此很容易面临胰岛素短缺。诸多国家在胰岛素上投入了大量资金，且成本在不断增加。某些国家缺乏胰岛素采购、供应链管理和分销所需的基础设施。将供应安全概念应用于胰岛素，表明需要促进供应商的多样化。全球卫生行动者应采取供应安全的方法来确定易受供应问题影响的药物，并通过战略性提升本地产量、加强监管协调，以及将本地产品纳入世卫组织药品资格预审项目来解决这一问题。

## Резюме

Надежность поставок лекарственных средств — основа обеспечения всеобщего здравоохранения. Кроме того, улучшение доступа к лекарственным средствам включено в цель номер 3 программы устойчивого развития. Однако концепция надежности поставок в основном применялась к пищевым продуктам, воде и энергии. Разнообразие источников поставок, уязвимость к сбоям поставок, затраты, инфраструктура, стабильность в странах-экспортерах, владение производством, стабильность цен, доступность и равные возможности доступа, приемлемая цена, вопросы интеллектуальной собственности, безопасность и надежность поставок и способность страны адаптироваться к изменениям на рынке — все это элементы системы обеспечения надежности поставок. На основании этих элементов авторы оценивали надежность поставок инсулина, так как доступность инсулина является глобальной проблемой. Было обнаружено, что три транснациональные компании (из Германии, Дании и Франции) совместно контролируют 99% мирового рынка производства инсулина. Цены, доступность инсулина и возможность его использования значительно различаются в разных странах. Некоторые страны весьма чувствительны к дефициту инсулина, поскольку они импортируют инсулин только из одного источника. Многие страны тратят огромные суммы денег на приобретение инсулина, и затраты на него постоянно растут. В некоторых странах отсутствует необходимая инфраструктура для закупок, управления цепочкой поставок и распределения инсулина. Применение концепции надежности поставок к инсулину показало, что необходимо способствовать диверсификации поставщиков. Организации, действующие в области мирового здравоохранения, должны внедрить у себя подход обеспечения надежности поставок, чтобы выявлять лекарственные препараты, подверженные перебоям в поставках, и решать соответствующие проблемы посредством стратегического развития местного производства, укрепления процессов согласования режимов регулирования, а также введения местной лекарственной продукции в программу Всемирной организации здравоохранения по предварительной квалификации лекарственных средств.

## Introduction

Improving access to medicines is included in sustainable development goal (SDG) 3, which is to ensure healthy lives and promote well-being for all at all ages.^1^ Nonetheless, estimations show that worldwide one person in three has no guaranteed access to the medicines they need.^2^ Progress on improving access to medicines has been made for vaccines, antiretroviral medicines for human immunodeficiency virus, medicines for tuberculosis and malaria, and contraceptives. However, access to medicines for noncommunicable diseases is still a problem.^3^ A study showed that generic medicines to treat noncommunicable diseases were less available than generic medicines for communicable diseases in both the public and private sectors in low- and middle-income countries.^4^

Availability and affordability of medicines in both the public and private sectors are important indicators of access to medicines. This relation has been recognized by the World Health Organization (WHO) in its *Global action plan for the prevention and control of noncommunicable diseases 2013–2020*, which includes a specific target on access to medicines of “an 80% availability of the affordable basic technologies and essential medicines, including generics, required to treat major noncommunicable diseases in both public and private facilities.”^5^

As a medicine to treat diabetes, a noncommunicable disease, insulin stands out because, despite having been discovered in 1921 and first administered to an individual in 1922, access remains problematic.^3^ This issue persists even though there are no patents on human insulin. A possible barrier to access is the dominance of three multinational companies^3^ and the fact that that old products have been replaced with newer ones, but no manufacturers of biosimilar generic products have emerged on the market.^6^

One factor that influences availability and affordability of medicines and other health technologies is that many countries must import them. Some have argued that this dependence on imports puts security of supply at risk.^7^ The literature on security of supply has focused on food, water and energy.^8^^–^^10^ The aim of this article is to present the concept of security of supply for medicines using insulin as an example.

## What is security of supply?

The concept of security of supply has its roots in the security of supply of crude oil and the link between this supply and military and economic development.^9^ Only some countries produce and supply crude oil, therefore there is a dependence on these countries. Oil is a global commodity, and prices can be volatile and are linked to the value of the United States dollar (US$). Increases in demand for oil in some areas of the world have a global impact on price, and there is a reliance on a vast cross-border infrastructure.^9^^,^^11^ To manage security of supply for crude oil, countries have diversified their sources of energy and built up national reserves.

Another area where the concept of security of supply has been used is food security. Researchers argue that the global food system is dysfunctional when a portion of the population does not have access to sufficient food and another part is overfed.^12^ They state that, because of globalization of food markets, increases in consumption of certain foods in some areas of the world have affected other areas of the world in terms of price and availability of certain foodstuffs. In addition, changes in factors that influence supply, such as availability of resources and climate change, affect what is supplied, to whom and at what price.

Several articles have discussed what security of supply entails. For example, authors have suggested that because of a dependence on external sources for key resources this can be seen as an issue of economic and national security.^10^^,^^11^ Several factors can affect the continuous supply and affordability of goods, such as interruption of supply due to, for example, war, sanctions and disruptions within the supply chain, and the monopoly of a foreign supplier, as well as technical, human, and natural risks.^11^ Assessment of these risks needs to take into account the availability of substitutes, how quickly the threat can affect supply, whether the threat will lead to constant or temporary scarcity, and the duration and magnitude of the interruption. 

For security of supply for medicines, the United Nations Children’s Fund (UNICEF) considers three essential components of supply: uninterrupted, sustainable and provides affordable, quality medicines.^13^ A report of the Department for International Development of the United Kingdom of Great Britain and Northern Ireland on global health partnership impact on commodity pricing and security^14^ presents the issue of security of supply for medicines and vaccines in terms of: (i) uninterrupted supply, meaning stock-outs do not occur because lead times and reserve stocks are sufficient; (ii) sustainable supply, meaning the market is attractive enough to maintain current production capacity and attract new producers to have a competitive market; and (iii) good quality supply, meaning medicines meet international quality standards.

Other elements, such as diversity of supply, vulnerability to disruption, amount spent on energy, infrastructure, stability of exporting countries, ownership of production, stability of prices, access and equity, affordability, intellectual property issues, safety and reliability of supply, and capacity of countries to adapt to changes in the market, can also affect the security of supply of oil.^8^ As such, the definition of security of supply goes beyond vulnerability of production to also encompass supply systems and infrastructure.^9^^,^^11^ We used these 12 elements to assess the security of supply for insulin below and our findings are summarized in Box 1.

Box 1 **Definitions of security of supply^8^ applied to insulin**

**Diversity of supply** – The market is dominated by three multinationals and other suppliers do not have a global reach.

**Vulnerability to disruption** – Some countries depend on one supplier for their insulin. Companies change the products available on the market.

**Expenditure on insulin** – The high cost of insulin affects health budgets and out-of-pocket expenditure for individuals.

**Infrastructure** – Infrastructure in many countries for procurement, supply chain management and distribution is weak.

**Stability of exporting countries** – Main countries exporting insulin (Denmark, France and Germany) are stable.

**Who owns production** – Most global facilities producing insulin are owned by three multinational companies and their shareholders.

**Stability of prices** – Prices of insulin vary between countries and are high. A variety of factors affect the price to the health system and the individual.

**Access and equity** – Access varies between and within countries depending on health systems and their coverage.

**Affordability** – Affordability is a challenge for patients and health systems. Insulin is purchased by ministries of health in low- and middle-income countries using domestic revenues not funds from donors. The shift to more expensive insulin analogues increases the challenges.

**Intellectual property** – There are no patents on insulin itself, but patents on delivery devices are increasing.

**Safety and reliability of supply** – Insulin requires a cold chain. Products from the multinational companies are safe and reliable. Some formulations of insulin have been removed from the market by manufacturers.

**Capacity to adapt to market changes** – Countries have very little capacity to adapt given limited suppliers and there is no alternative for people with type 1 diabetes.

## Applying security of supply to insulin

### Diversity of supply

The first element is the diversity of supply.^8^ For insulin, the global market is dominated by three multinational companies which control 99.0% of the value of the insulin market and 96.0% of its volume.^15^ The remainder of the market is shared by 42 other insulin manufacturers in 17 countries.^16^ This domination of the market can also been seen in trade reported in the United Nations (UN) Comtrade database.^17^ For exports, from 2003 to 2013, 10 countries made up between 97.9 and 99.0% of the global value of retail insulin exports. Denmark, France and Germany together exported 84.6–95.8% of global retail insulin by value over this period.^18^ In these three countries, the three multinational companies manufacture insulin. Looking at product registrations, 88.7% of global insulin registrations are for products from the three multinational companies.^16^

### Vulnerability to disruption

We also assessed vulnerability to disruption of the supply of insulin. About 60 countries, mostly low- and middle-income countries with no local insulin manufacturing, imported insulin from only one country for at least one year. These data also show that only 17.4% (8/46) of sub-Saharan African countries purchased insulin every year during 2004–2013. Worryingly, 26.1% (12/46) of sub-Saharan African countries did not report buying insulin at all over this period.^18^ In addition, there is no generic or biosimilar insulin on the market because insulins are many different products depending on, for example, brand, type of action and formulation.^6^ Furthermore, as new insulin formulations appear, older formulations simply disappear from the market and are not replaced by generic products. At the same time, because of the domination of the three companies, some formulations have been withdrawn from the market.^19^ Globally, animal insulin has almost completely disappeared and been replaced by human insulin. Now, human insulin is being replaced by insulin analogues, despite a lack of evidence that it is more effective than human insulin, and its higher cost.^20^

### Expenditure on insulin

Many countries spend a large amount of money on insulin and these costs are increasing. Insulin purchases in Mozambique in 2003 represented as much as 10.0% (US$ 603 824/6 000 000) of government expenditure on all medicines.^21^ Data from five European countries in 2010 show that spending on diabetes medicines (insulin and oral medicines for diabetes) was between 6.2% (France and Italy) and 10.5% (Spain) of total direct cost of diabetes care.^22^ In addition, between 2005/2006 and 2015/2016, diabetes expenditure doubled in the United Kingdom of Great Britain and Northern Ireland.^21^ The long-acting insulin glargine was second in terms of expenditure on medicines in government-funded programmes in the United States of America (USA). In addition, in the past 20 years, increases in diabetes expenditure in the USA have mainly been the result of higher spending on medicines.^21^ Finally, based on 11 countries where patients must pay for their medicine, the average annual cost of insulin to the individual was US$ 35.40 in the public sector and US$ 95.71 in the private sector.^20^

### Infrastructure

Many countries lack an adequate infrastructure for procurement, supply chain management and distribution of insulin, which results in low availability of insulin. Lack of these systems can cause problems such as quantifying needs at the national level, in-country distribution, and determining needs at lower levels of the health system.^20^ For example, in Mozambique, although Maputo Province represented only 11.3% (2.0/17.7 million) of the total population of Mozambique in 2003, the province received 77.3% (46 130/59 657 vials) of the total amount of insulin purchased by the health ministry. In Kyrgyzstan because of weak planning and distribution systems, health facilities received only what was available at the central level rather than what they had ordered.^23^

### Stability of exporting countries

The stability of exporting countries and who owns production are also elements of security of supply.^8^ For insulin, the main export countries are Denmark, France and Germany, and they are relatively stable.

### Ownership of production

Most of the global insulin production facilities are owned by three multinationals in these countries and their shareholders, and there are 42 smaller insulin manufacturers.^16^

### Stability of prices

**Fig. 1:**
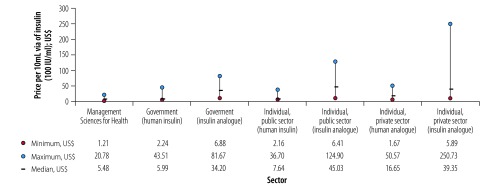
Prices of insulin to government and individuals in public and private sectors based on data from various countries

Note: Management Sciences for Health is a global non-profit organization, partnering with governments, civil society, the private sector, and health-care workers to build resilient and sustainable health systems. Their data are from government tender prices for 1996–2013 and standardized at 2015 prices.

Source: Ewen M et al.^24^

Prices of insulin vary between countries and prices are high. Several factors affect the price for the health system and the individual. These factors include the increasing market share of insulin analogues. Fig. 1 shows that prices differ considerably for the same insulin formulation. Based on data from several countries, the highest prices for insulin analogues are 12 times higher than the lowest price in the public sector and 43 times higher in the private sector.^24^ Furthermore, the median price per vial for insulin analogues for governments, and patients in the public and private sectors is 5.7, 5.9 and 2.4 times higher respectively than for human insulin.

As many countries buy their insulin in US$, this may affect its actual cost in local currency. For example, in Nicaragua the price of insulin to the government in 2005 was 98.05 cordobas (US$ 5.93) per vial with a total expenditure on insulin of 6 661 734 cordobas (US$ 399 283) for 67 459 vials. In 2006, the price per vial was on average 91.03 cordobas (US$ 5.06) for a vial of neutral protamine Hagedorn (NPH) insulin and 96.67 cordobas (US$ 5.37) for a vial of rapid-acting insulin (human). Total expenditure on insulin in 2006 was 9 005 427 cordobas (US$ 500 302) for 94 351 vials. This represents a 39.9% increase in the quantity of insulin from 2005 to 2006, but a 35.2% increase in cost in cordobas (25.3% increase in US$).^25^

### Access and equity

Access and equity between and within countries varies depending on the organization of the health system and the health-care coverage. The availability of insulin at the facility level is poor in many low- and middle-income countries, ranging between 17.4% and 100.0% of facilities in the public sector (median: 42.9%) and 0.0% and 95.0% in the private sector (median: 35.0%).^20^ Equity is also an issue at global and national levels. Globally, one in two people with type 2 diabetes has access to the insulin they need, but in Africa this number is one in seven people.^26^ In some countries in sub-Saharan Africa, insulin was more available in higher levels of the health system (e.g. in hospitals rather than health-care centres) and in the private sector than the public sector.^27^

### Affordability

Unlike many other medicines and vaccines, insulin is purchased by health ministries in low- and middle-income countries using domestic revenues, rather than funds from bilateral or multilateral donors. Affordability is a problem for patients and health systems, and this challenge is increasing with the shift to more expensive insulin analogues.^20^ Insulin remains unaffordable to many people and countries. For example, in Zambia, the annual cost of insulin for the individual in 2003 was US$ 26.00 in the public sector and US$ 218.40 in the private sector, representing 2.7% (US$ 26.00/953) and 22.8% (US$ 218.40/958) of per capita nominal gross domestic product. These figures mean that the lowest paid government worker in 2013 needed to work less than one day if the insulin was available in the public sector or 9 days if they needed to buy their insulin in the private sector. However, even US$ 26.00 a year for insulin might be unaffordable in a country where 69% of the population lives below the international poverty line of US$ 1.25 a day.^20^ Insulin is also an increasing financial burden on health systems that provide insulin for free to their populations.^21^

### Intellectual property

Both process and product-related patents for insulin need to be considered. Intellectual property would not seem to be a barrier to entry into the market as most patents on human and analogue insulins have expired or will be expiring.^28^ However, an increase in patents on delivery devices has been seen.^29^

### Safety and reliability

The safety and reliability of the products of multinational companies do not seem to be issues as they have been approved by stringent regulatory authorities. As some formulations of insulin have in the past been removed from the market by the manufacturers, this may pose reliability issues for individuals accustomed to using those specific products;^19^ for example, the disappearance of animal insulin in favour of human insulin. Current trends suggest that such a change might be happening for human insulin, which is being replaced by insulin analogues.^20^ Insulin also requires a cold chain, which has not been identified as a major problem within the health system, but could be a problem at the individual’s home.^20^

### Adapting to market changes

Recent data have shown a gap between current access to and need for insulin.^26^ In 2018, estimations showed that 30.2 million people were able to access the insulin they needed, whereas the actual need was more than double, with 63.3 million people needing insulin. If comprehensive insulin access were available globally, about 80 million people would require access to insulin in 2030. The data also show an increase in the need for insulin vials of 22.7% (1716/5161 million 1000 international unit (IU) vials) over the period 2018 to 2030.^26^ Several factors that affect access to insulin need to be improved. Security of supply offers a different way to consider access to medicines. Current and future production capacity of insulin is unknown, thus it is unclear if manufacturers will be able to meet future demand. Importantly for type 1 diabetes, insulin is the only treatment that can be used. Given the limited number of suppliers, the capacity of countries to adapt to market changes may be in question.

## Discussion

Access to safe, effective and good quality essential medicines, vaccines and medical products is crucial to achieving universal health coverage and the health-related targets of the SDGs.^1^ The issue was again highlighted at the World Health Assembly in 2018 with discussion on the global shortage of, and access to, medicines and vaccines.^30^ Specifically for noncommunicable diseases, challenges related to rising prices, shortages and stock-outs were emphasized. WHO was requested to prepare a road map on this issue for presentation to the World Health Assembly in 2019.^30^

Several characteristics of insulin make its manufacture and supply a challenge, regardless of where manufacturing takes place. Unlike other pharmaceuticals, insulin is a biological product and is injectable. These characteristics have implications for the cost of upfront investments in manufacturing, the scale of production to reduce manufacturing costs and the cost of goods, maintenance of good manufacturing practices and quality control standards, and regulatory approval processes. These factors affect the numbers of producers as well as access to markets for smaller companies.

Safeguarding the supply of good quality essential medical products is fundamental to ensuring health for all. This issue also concerns high-income countries which have produced government reports and recommendations on the security of drug supply.^31^^,^^32^ In applying the concept of security of supply to medicines, a greater diversity of suppliers needs to be fostered by promoting competition through generic products. In applying the concept of security of supply to these products, the diversification of suppliers needs to be fostered by promoting generic competition. Global health actors, including WHO, need to adopt a security of supply approach to identifying medicines that are susceptible to supply problems, and address the issue by strategic promotion of local production, strengthening harmonization of regulations and adding those products to WHO’s programme on prequalification of medicines to effectively deal with possible shortages and other access challenges, which have been seen even in high-income settings.^33^

With the increase in the prevalence of diabetes and need for insulin,^26^ and the limited sources of supply, smaller markets may suffer. For example, some Asian pharmaceutical companies are prioritizing the more lucrative markets of high-income countries, which may affect the range of products available in Africa.^16^ Although for crude oil and other products, a global stockpile might be a solution, because insulin needs to be stored in a cold chain and has an expiry date, a global stockpile may not be appropriate for insulin and other medicines. The price of insulin is one aspect of the complex issue of access to treatment and management of diabetes. A variety of factors related to health systems also have a great effect on a patient’s access to diabetes treatment. These factors include appropriate levels of financing for treatment, adequate medicine procurement processes, access to diagnostic tools, trained health professionals, organized health-care delivery, and an overarching policy response for noncommunicable disease, including specific measures for access to medicines.
